# A systematic review and qualitative evidence synthesis of factors affecting mHealth adoption in India

**DOI:** 10.1093/oodh/oqae046

**Published:** 2024-11-19

**Authors:** Verghese Thomas, Judy Jenkins, Jomin George

**Affiliations:** Health Data Science, Swansea University, Singleton Park, Swansea, SA2 8PP, Wales; Division of Medical Informatics, St John’s Research Institute, 100 feet road, John Nagar, Koramangala, Bangalore, 560034, India; Health Data Science, Swansea University, Singleton Park, Swansea, SA2 8PP, Wales; Health Data Science, Swansea University, Singleton Park, Swansea, SA2 8PP, Wales

**Keywords:** mHealth, adoption, implementation, India, qualitative synthesis

## Abstract

mHealth implementations are increasing in low- and middle-income countries to strengthen health systems and improve health outcomes. Following the proliferation of mobile internet use, Indian health systems have deployed mHealth widely. However, there is little evidence that mHealth has improved health outcomes in India across settings and at scale. The aim of this study was to review current evidence on perceptions and experiences of end users of mHealth in India and synthesize qualitative data to determine the factors influencing mHealth use to inform mHealth design, development, and implementation. A systematic review and qualitative synthesis of studies on mHealth in India was conducted by searching the Web of Science, Medline and CINAHL databases for qualitative studies on mHealth users including both health system beneficiaries and healthcare personnel. Findings from the studies were synthesized using thematic synthesis. The synthesis generated the themes of the Environment, the Users and the mHealth system. The data indicate that mHealth use improves when the environment supports its use; when users are motivated and have the ability to use mHealth systems; and when mHealth systems are aligned with the environmental context and fulfill users’ needs and desires. mHealth adoption in India can be improved through human centered design and by addressing the disparities in digital literacy between socio economic strata. These approaches are required to close the design reality gaps facing mHealth systems, to improve mHealth implementation for health system strengthening, and therefore, to improve health outcomes in India.

## Lay Summary

Several mHealth interventions have been deployed for health promotion in India but they have not resulted in tangible benefits when scaled. The findings of recent implementation research on mHealth in India need to be synthesized to inform current and future mHealth design, development and implementations. A systematic review was done to find qualitative studies on mHealth in India and the data from selected studies were synthesized. The data synthesis found that alignment between the environment, users and mHealth systems has influenced the perceptions and experiences users in India have with mHealth systems. This synthesis explores these influences in Indian contexts and their interrelationships. This study identifies design reality gaps between mHealth systems in India and their environments and users that need to be addressed to improve mHealth adoption. This study suggests these gaps can be overcome by contextualizing mHealth systems through human-centered design, by providing effective system support and training, and by addressing the digital divide in India.

## INTRODUCTION

Although mobile applications have been deployed widely for health system strengthening in India and similar countries, additional measures are needed to ensure they achieve sustainability and impact at scale. In recent years mHealth systems have been increasingly used in low- and middle-income countries (LMICs) including India for public health interventions and strengthening health systems [1, 2]. In 2020, over 13 mobile apps were deployed to support Indian national public health programs [3] and more have been added after the COVID-19 pandemic. However, there is a dearth of evidence that mHealth systems in LMICs have achieved scale and sustainability [1] or provided wide spread benefits to health systems [4]. Several studies have reported significant barriers to using data from health information systems in LMICs [5]; and that there are challenges to mHealth system sustainability in LMIC settings [6]. Similarly in India, there is little evidence of benefits from mHealth at scale. A recent systematic review and metanalysis of mHealth interventions in India found that while individual disparate studies on mHealth indicated improved health outcomes, a metanalysis of comparable studies demonstrated significant improvements in only one of three process measures investigated [7]. This is despite the fact that some of the world’s largest digital health platforms are deployed in India [8, 9].

The nature of system use has a direct bearing on the benefits acquired from an information system [10] and is heavily influenced by users’ perceptions and experiences [11, 12]. Therefore, the influences on mHealth use in India, as perceived by its users, need to be explored to generate evidence to inform policy to improve mHealth adoption in India. The objective of this study is to synthesize qualitative evidence of the perceptions and experiences of healthcare providers, patients, and other health program beneficiaries that influence mHealth adoption in India.

## MATERIALS AND METHODS

A systematic review of qualitative studies on mHealth in India was done in August 2023, followed by a qualitative synthesis.

### Eligibility criteria

Qualitative studies on health program beneficiaries and healthcare providers in India were considered. Healthcare providers included community health workers (CHWs) and any health facility personnel. The intervention studied was mHealth, defined as any system which used mobile technology for health and health-related fields. The outcomes were perceptions and experiences of mHealth. Only studies that used qualitative methods to collect and analyze data were included. Mixed methods studies were included if qualitative data could be extracted from them.

The review was limited to English peer reviewed articles published from January 1, 2018, to July 31, 2023. The review was limited to 5 years because growth in mobile internet use in India stabilized after 2017 [13].

### Sources of information

The Web of Science, Medline, and CINAHL databases were searched.

### Search

On 1st August 2023 the topic field in Web of Science and titles or abstracts in CINAHL and Medline were searched with the following terms:

(‘mhealth’ or ‘mobile health’ or ‘m-health’ or ‘health app^*^’ or ‘digital app^*^’ or ‘mobile app^*^’ or ‘smartphone^*^’ or ‘smart phone^*^’ or ‘mobile phone^*^’ or ‘tablet^*^’) AND.

India^*^ AND.

(‘qualitat^*^’ or ‘action research’ or ‘document analysis’ or ‘ethnographic research’ or ‘ethnological research’ or ‘ethnonursing research’ or ‘grounded theory’ or ‘phenomenological research’)

Any papers found incidentally after the search were added to the search results.

### Study selection

The search results were uploaded into the Covidence platform [14] where duplicates were removed automatically and manually. Studies were screened by title and abstract, and then by full text against the inclusion criteria for inclusion in the review. The screened studies were appraised against the Critical Appraisal Skills Programme Qualitative Studies checklist 2018 [15]. Rigor in data collection and analysis; and conflicts of interest were the primary criteria used to select studies for synthesis. One reviewer (VT) conducted initial screening, appraisal, and selection of studies, which was cross-checked by two other reviewers (JJ and JG) to ensure consistency.

### Data collection

The studies selected were analyzed with NVivo (release 1.7.1). The results sections of selected articles were used as the data of the synthesis as per thematic synthesis methodology [16]. The results and findings were extracted by one reviewer (VT) and cross-checked by the others (JJ and JG).

### Synthesis methods

The studies were synthesized using thematic analysis [17] and thematic synthesis [16]. First thematic analysis was used. Studies were read and annotated for familiarization, the results and findings were then coded line by line using semantic open inductive coding to tag text sections with code labels for each meaning in the extracts that were relevant to the research question. Granular descriptive codes were generated and grouped into higher level codes. Using thematic synthesis, initial themes and subthemes were determined and mapped using analytic theme generation to provide higher level interpretations of the texts.

The perceptions and experiences in the data were interpreted through technology adoption theories [11, 12], information system theory [10], and frameworks for health IT system constructs [18] to generate central organizing concepts and the relationships between these concepts that explained the use of mHealth in the data. The themes were developed and revised by reviewing the coded extracts and revised again by reviewing the entire data set. The coded extracts were reviewed and reorganized into a three-level hierarchy while the themes were being developed and revised. The themes were then refined, named and theme descriptions were written. The final list of codes consisted of 603 unique codes in three levels with 43 higher level codes. The themes, subthemes and relationships were mapped and abstracted into a higher order thematic map. The analysis was conducted by one reviewer (VT) and cross-checked by the other reviewers (JJ and JG).

Patients and the public were not involved in the design and conduct of the study.

## RESULTS

### Study selection

**Figure 1:**
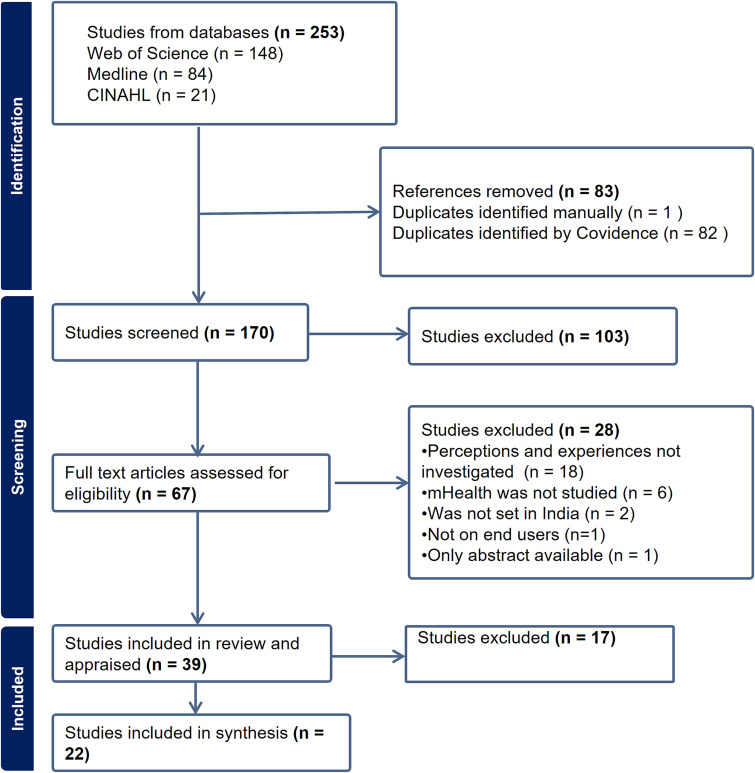
PRISMA diagram of study selection

The electronic search returned 250 articles. Three additional articles were found incidentally. 83 duplicates were removed. 170 titles and abstracts were screened, out of which 67 studies were assessed for eligibility from the whole texts. 39 studies were included in the review and appraised for quality. 17 studies [19–35] were excluded through the appraisal and 22 studies constituted the synthesis dataset. The selection of studies is illustrated in the PRISMA diagram in Fig. 1. Descriptions of the studies examined are in Appendix 1 and the appraisal findings are in Appendix 2.

### Synthesis results

**Figure 2:**
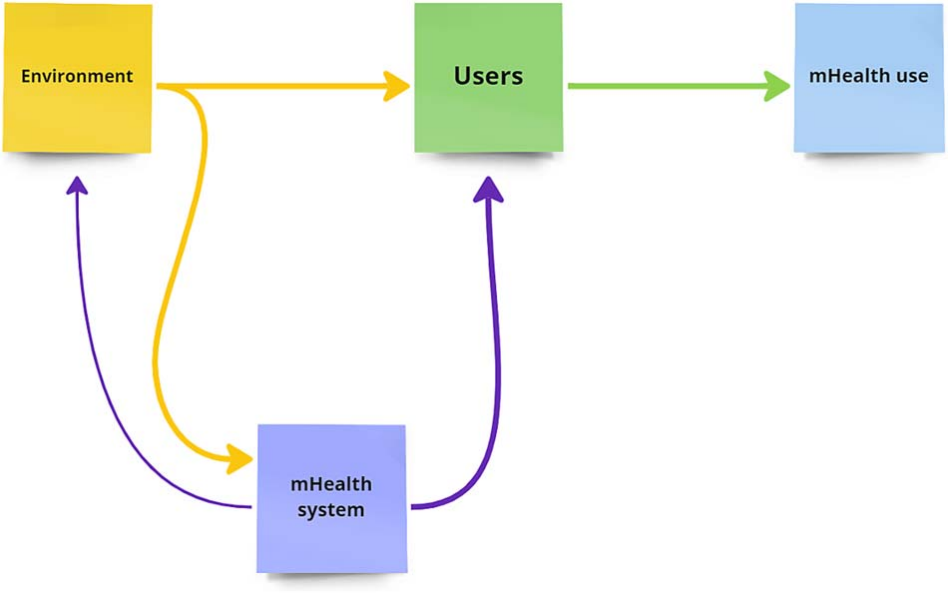
Themes of influences on mHealth use

Analysis of the data from the studies selected indicate that there are three principal themes that influence the adoption of mHealth systems in Indian settings. The themes are the Environment, the Users and the mHealth solution. The interplay of these themes influences mHealth use. The data explores the facets and relationships between these themes in Indian contexts as summarized in Fig. 2.

### The environment

There were perceptions and experiences of environmental phenomena external to mHealth systems and users which influenced mHealth use.

#### The Mobile network and electricity

Poor quality mobile networks significantly hindered mHealth systems used by CHWs [36–39] and patients [40] in both rural and urban settings [41]. Network problems affected program implementation as phones would freeze while being used with patients [36] and data transfer was interrupted [42, 43]:

“ ‘Signals…very poor at time. Sometimes they [the data] are not synchronizing. So many times, we faced inconvenience because of this synchronizing process. The data collected by ASHA’s did not synchronize into our tabs. It took so many days for that [to be sorted out]’. (Doctor, IDI-4)” [43].

Network pricing affected mHealth use. In some cases the network problems were due to increasing rates or payment delays [44, 45]. In another setting, the introduction of free outgoing calls by a service provider led to more calls between CHWs and community members:

“…our participants shared that access to free outgoing calls due to Reliance Jio had helped them build a better rapport with low-literate communities. Before Jio, outgoing calls were charged resulting in many community members not calling at all or doing missed calls; residents were more likely to call ASHAs now.” [41]

A lack of electricity at health facilities and homes was also cited as a hindrance to mHealth use by CHWs [36] and patients:

“I am staying in a hut, so I don’t have electricity in my home; we burn [wood] sticks to get light.” (late 40s male, HIV coinfected, on treatment for tuberculosis) [46]

#### The health system

Health systems, including users’ peers, influenced mHealth use. Some health programs failed to recharge Subscriber Identity Modules leading to loss of mobile network access [39]. Health systems affected the motivation of their staff to use mHealth solutions. Some CHWs felt motivated to use an mHealth system because of pressure from their supervisors, fears of consequences on their employment, and anticipated rewards [45]. Elsewhere, health program staff felt demotivated because they were not reimbursed for mobile network costs:

“We should get the reimbursement of the call charges. Otherwise the motivation to work will be affected.” (male counsellor, early 30s) [47].

The hierarchical structure and culture of Indian health systems extended into mobile online forums for healthcare teams [41, 48], and affected participation:

“…maybe a [nurse with 20 years of experience] might not open up because if she had done any mistake then she is under fear that everybody will get to know about that” [48]

With respect to peers in the health system, some CHWs drew support from their peers in learning to use a system and for trouble shooting [49, 50].

With regard to patients’ motivation, the treating clinician was viewed as an important influence on patients and their families for promoting an mHealth application.

“...the caregivers said that the treating doctor is needed to promote an active positive perception towards the app. If treating clinicians do not emphasize the importance of such apps, patients and family members are generally discouraged from using them.” [51]

#### The community

Attitudes and practices in communities affected the way mHealth was used by CHWs and beneficiaries. In some communities, CHWs were unable to use mHealth systems to support counselling because they were not allowed to meet pregnant women [36] or pregnant women migrated to their parents’ homes, following traditional practices [36, 38].

“If in case of Rajput community, they do not send brides out. Tell to their household members whatever you want to convey. They let do but only when there is no one at home or only female members are at home. They do not let them come out as if they would go out somewhere. . .. I go to such place sometimes and explain and tell them. Rest is their wish, we can’t force them.” (Anganwadi worker, Madhya Pradesh, early 20s, 12th grade education)” [36]

Prevalent social norms and practices also had a significant effect on the acceptance of health information from mHealth systems. For some mHealth solutions, health information was only accepted if it was in line with existing community norms, as conveyed by elders in the family:

“I: Between the two who was right? Your mother-in-law was right or what you heard from doctor Anita in the phone call was right?

R: I felt my mother-in-law was right.

I: Your mother-in-law was right. For what reason?

R: Because she’s elder so she knows. The others don’t know much.

I: And what Doctor Anita is saying what do you think that you should follow her instructions or not?

R: We should do it.

I: Okay, for what reason you didn’t do it?

R: When the elder said so I had to do it. (WOM_23)” [44]

However, mHealth systems were considered the most important sources of health information for mental health [51] and sexual health issues [52].

“Information regarding the nature and course of illness, the treatment and management of side effects, vocational rehabilitation, emergency services, government benefits and schemes, ambulance, daycare centers, and NGOs were highly sought after. Although such information is available on the internet, many caregivers wanted information from sources they could trust. Internet results on mental disorders and its treatment were often in conflict…” [51]

These differences in acceptance could be because of socio economic differences between the beneficiaries of the mHealth solutions and also because of differences in the availability of information that was considered reliable:

“ ‘Patient-related information is available on the Internet, but I have spent nights after nights in search of information on how a caregiver can handle patient[s] in certain situation, there is no data available for us.’ [F1C3]” [51]

The social environment also affected mHealth use through social stigma. This affected mHealth systems for sexually transmitted diseases [52], tuberculosis, and HIV [40]:

“I am very worried about my children coming to know [about the TB diagnosis]... so I am making calls while hiding from others,” (early 50s male, HIV coinfected, on treatment for tuberculosis). [46]

#### The family

Families played an important role in supporting mHealth use by CHWs [53] and patients [40, 54] as well as discouraging mHealth use [55]. Family dynamics in rural households determined the effectiveness of mHealth use. mHealth interventions for maternal and child health targeted women in rural households but their mothers-in-law determined child rearing practices [44] and in the majority of households, husband controlled their access to mobile phones [37, 43]. In the rural households studied, phone ownership was often restricted to the husband, so beneficiaries’ and in some cases, CHWs’ access to mHealth was contingent on their husband [45, 50].

“ ‘Whenever I say that I have to take it to work, he gives it to me. When he wishes to use this phone, I leave it at home and ask him to operate it, as I would have no use for it on that particular day. I do not take it. If I need any information I come back [home] and see it.’ ” [50]

There were instances when husbands prevented the intended beneficiary from receiving an mHealth intervention:

“ ‘R: Sometimes he tells that a call had come from Kilkari. […]

I: Does he tell what they told him?

R: No, he doesn’t tell that. […] When I ask him [about the call], he says, ‘Nothing, it was just like that’. […] He is not that interested. […] Since he is mostly driving, he doesn’t get the time.’ ” [44]

In other settings, phone sharing in the family caused errors in data entry [46] and breeches in confidentiality.

“ ‘When the patient has to be contacted through someone else in the family (when patient does not own the phone), we have to first explain in detail to reach the client. In such cases, we may have to break the confidentiality.’ (female counsellor, early 30s)” [47]

The data demonstrated the influence the environment exerted on mHealth use in Indian settings, as perceived and experienced by mHealth users. mHealth use was determined by the effect of these influences in combination with features of mHealth solutions and influences arising from the users themselves.

### The users

mHealth system users were either beneficiaries of public health programs or healthcare personnel working in community and clinical settings. The data indicates that determinants arising from the users themselves influenced mHealth use. The effect of the environment on mHealth use was moderated by users’ access to mobile technology; digital literacy; needs, desires, and fears; and users’ experiences of being helped or hindered by mHealth.

#### Users’ access to Mobile technology

As mentioned above, women in rural households often did not have their own mobile phones and their access was mediated by their husbands [37, 44]. Among some women CHWs, access to the internet was irregular and influenced by their husbands who owned the smart phones and were members of the CHW WhatsApp groups, relaying information to the CHWs [45, 50]. These findings indicate the effect of prevailing gender disparities on mobile technology access and mHealth use in Indian rural communities.

Users’ ability to afford mobile phones and internet access influenced mHealth use. Some caregivers of mental health patients had reservations about affordability:

“According to most caregivers, lack of money either to own a smartphone or an active Internet connection was a major problem.” [51]

Elsewhere, a hike in mobile recharge tariffs forced women to stop using their own phones and instead share their husbands’ phones.

“…several women shared they could not dial in to IVRS on certain days as the phone was with husband. With a hike (0.5$/month) in recharge tariff, these underprivileged women have slowly adapted themselves from using their own phone to sharing their husbands’ phone.”

Among urban hospital nurses however, there was universal access to mobile internet networks [48]. This is probably due to the socio-economic differences between these users and those described earlier.

#### Users’ ability to use mHealth

Users’ capabilities in using mHealth systems influenced mHealth use. Some beneficiaries and CHWs with lower qualifications were unable to type on mobile phones [38, 45], were unable to use interactive voice response systems (IVRS) [43], had difficulty with reading text [51, 52, 55] and were generally unfamiliar with smart phones [38, 54]. This was ascribed to being from rural areas, age, literacy and education, income, and language spoken [41, 56].

“ ‘There are so many ASHAs (a type of CHW) on this group but no one responds. . . I understand that some ASHAs have trouble typing so they don’t respond, they just read. Others at least, please respond.’ (P3)” [41]

A lack of digital literacy reduced some CHWs engagement with online groups with colleagues, leading to their marginalization [41]. These CHWs developed workarounds to overcome their difficulties in typing using features available on their phones such as speech-to-text and emojis.

#### Users’ needs, desires, and fears

Studies on mHealth use elicited perceived needs, desires and fears which influenced intentions to use mHealth. Some needs, desires and fears arose from the users’ environment as explained above. But there were also motivations to use mHealth which arose from the users.

The need for the provision of health information from mHealth systems was expressed in formative studies on mHealth for mental health and sexual health [47, 51, 52, 54] and a study on maternal and child health [45]. Beneficiaries and healthcare providers requested information about their conditions, medication, and the availability of healthcare services:

“Also some messages on what food to eat, what exercise to do and some basic health-related information should be given by them.” (PLHIV (Person Living with HIV), male, 28 years)” [47]

There were also needs for specific functions*—*reminders, social networking with other patients or community members, patient monitoring, medical records, navigation to health services, and telemedicine services [51, 52, 54].

Caregivers of patients with severe mental illnesses had apprehensions about technology use by the patients under their care. They were afraid of excessive screen time, damage to expensive phones, unsupervised internet access, and making inappropriate phone calls. Some caregivers didn’t allow patients access to smart phones because of these concerns [51].

#### Users’ experiences of being helped or hindered by mHealth

The data indicates that users, particularly healthcare providers, experienced being helped or hindered by mHealth in achieving their goals. Healthcare personnel felt mHealth systems made work easier and more efficient [36, 38, 46, 47, 50, 55]; improved communication between healthcare personnel [48]; improved patient monitoring [46], improved health education of community members [38, 42, 47], and improved patient management [43, 49]. Perceived effects on beneficiaries included better medication adherence [57], improved mood [37] and learning life skills [58]. Patients and community members found health information from mHealth [37, 44] and medication reminders [40] useful. There were also negative experiences by healthcare providers such as an increased work load [46] and disturbed work-life balance [12, 13]. Some participants also expressed skepticism about the impact mHealth could have [44, 54].

The impact mHealth had in supporting community engagement by CHWs resulted from the interaction between CHW-community relationships [36] -

“The underlying nature of the AWW-beneficiary relationship influenced the extent to which CAS (an mHealth solution) enhanced the beneficiary-CHW interface.”

- and enhanced communication afforded by mHealth [41]:

“Our participants leveraged social media to assist in the development and maintenance of relationships with residents…our participants shared that access to free outgoing calls due to Reliance Jio had helped them build a better rapport with low-literate communities.”

mHealth affected healthcare providers themselves and in some settings enabled them to influence the environment. CHWs used mHealth to enhance their credibility [38] and legitimacy [41] in the community and felt it increased their status

“We feel proud [to use mobile phones], it’s a new way [of doing our work]. Even others must be impressed that we’re doing it [our work] on the phone.’ ” (Nurse) [53]

Healthcare providers were empowered by mHealth through improvements in their skills [45], knowledge [38, 48, 49] and confidence [38, 45, 49, 53]. Data capture on smartphones and communication through social media empowered healthcare providers to challenge hierarchies through collective action against health systems [41] and as individuals confronting hierarchies in clinical settings [48]:

“ ‘Two days ago, one baby’s x-ray was bad we took the picture and sent because I thought the senior resident [junior doctor] does not understand because he was new. He told me the x-ray is fine but I took the picture of the x-ray and sent it to sir [senior doctor] and I told him that x-ray is not fine, do something. The video making thing is helpful because at least you are having some proof that you are telling right.’ (Nurse)” [48]

The data explored the extent to which mHealth systems can assist CHWs. For behavior change communication and mLearning, in situations where CHWs were not well embedded, and for mLearning interventions where trainers were discouraging, mHealth solutions improved community engagement [36] and learning [45]. However, the systems could not compensate for poor CHW-community relationships or replace in person teaching, indicating that mHealth solutions were at best considered complementary to CHWs and trainers and not a substitute for them.

Similarly, CHWs believed that artificial intelligence for diagnosing conditions through mHealth apps cannot replace CHWs in providing care, but could support them:

“How will it take my place? It’s a machine. I just think that when we get it, we can work more efficiently. Instead of replacing us, it will create more place in the field for us.” [50].

CHWs believed that mHealth cannot provide the human interaction required to gain beneficiaries’ trust [50]:

“…we are present there in front of the parent. They appreciate that we are explaining things to them and talking with them, so they start trusting us. Mobile phones do not get the same level of trust. Parents have a higher degree of trust on the person in front of them, no doubt. The app have an advanced technique, but still I would say that the level of trust is not as high…” [50]

The findings above demonstrated the influence that factors arising from users themselves had on mHealth use. As described earlier, these factors were themselves influenced by the various aspects of the environment. In addition to these two themes, mHealth use was also determined by the effect of these influences in combination with features of mHealth systems.

### The mHealth system

The data indicated that features of mHealth systems and their implementations influenced acceptance and use through alignment with environmental and user-related influences.

#### Usability of mHealth systems

There were positive experiences of mHealth being easy to use [42, 44–46, 49, 53, 54, 58], enjoyable [45, 47], and useful [44, 49, 56].

“CHW, Padhar: ‘Since it’s step-by-step on the phones, it’s easy to ask all of the questions step-by-step using the phone. We can double-check each question before sending (if we’ve made any mistakes or left any questions blank), we revise all questions before sending. We get the patient ID SMSs immediately’ ” [53].

There were also experiences of systems involving redundant tasks [56],being difficult to use [42, 46], confusing [40], cumbersome [40], annoying [40, 41], and monotonous [46].

“[I am] tired of calling daily.” (late teens male, HIV uninfected, on treatment for tuberculosis) [46]

There were experiences of stigma induced by mHealth when devices were indiscrete [40].

“ ‘When I carry the box when leaving the health center, people know that I have TB. This is embarrassing, so I try to hide it, but it is too big.’ (Patient, male, aged late 30s)” [40]

There were perceptions of what makes mHealth systems effective. Simplicity and ease of use [54], gamification through levels and rewards for compliance [51, 52], and the use of audio visual media to deliver information [51, 52, 59] were requested from users. The language used in a system was considered crucial. Participants wanted systems to use simple non- technical wording [44, 54] in local languages [37, 38, 42, 44, 46–48, 59].

“ ‘I don’t know how to read, it should be in Odia!’ (Male tobacco user, IDI)” [59]

There were conflicting perceptions about the emotional tone of warnings conveyed through apps between beneficiaries and healthcare providers. For an app on sexually transmitted infections, healthcare providers wanted the app to incite fear through graphic images but the beneficiaries disagreed [52]. On the other hand, for a tobacco cessation app, healthcare providers wanted to avoid inciting fear but tobacco users requested graphic images to induce fear among addicts [59]. Given the variability in perceptions about mHealth messaging, systems have to be designed specifically for context and with the intended users, to be accepted.

There were also technical problems that inhibited system use such as malfunctions [38, 40], battery problems and screen size [42], phones heating [36], and durability of materials [40].

#### Characteristics of health information

Health information conveyed through systems need to be in short messages that are contextualized to beneficiaries in terms of topics covered, timing and frequency [59]. Health information was considered more effective if it was delivered through narrative stories. Beneficiaries appreciated content based in stories [58], requested stories of role models [52], and wanted the ability to share their personal stories with other patients [51].

#### Privacy protection

Users were very concerned about keeping their health information private off-line but were not as concerned about their information online. Social stigma led to user resistance when a system could ‘out’ a beneficiary to persons who could see the mobile phone or hear alarms:

“ ‘You should just have a logo and that’s about it. Logo or the identity interface should not have a gay feel… And if there is a way that a person could hide the app, saying like you have this antivirus software where when it gets camouflage with something else.’ (MSM) [Men who have Sex with Men]” [52]

“Suppose that my relatives visit my home. The box’s alarm could ring in front of everybody.... They may come to know that I have this disease. I would be so embarrassed in front of them. So, I don’t like this box.” (Patient, female, aged late teens) [40]

In contrast, personal information was considered secure if it was in an online community [52] and beneficiaries were willing to share their location data to receive benefits from an app, provided they had control over the access granted to the app [54].

#### Support for users from the mHealth system

Experiences and views on support provided were variable. Some implementations were effective in enabling users to overcome challenges in using systems [42] while others were not able to convey the purpose of a system and how to use it [40, 45]. Support was considered necessary to enable users with low technology capability to use systems [51].

**Figure 3:**
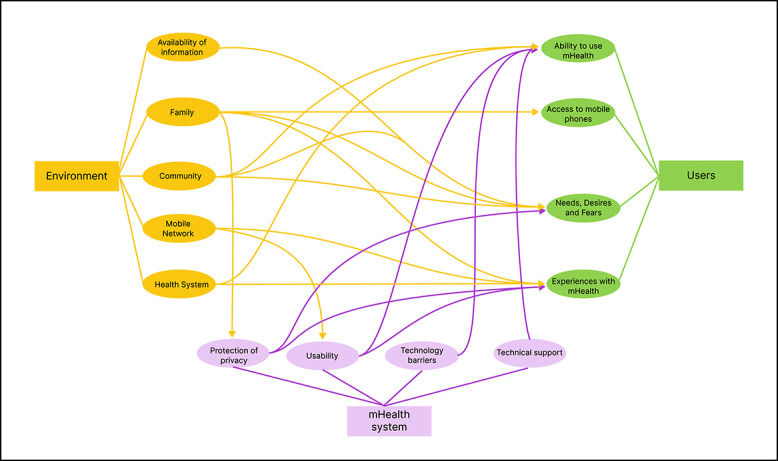
Themes and relationships that influenced mHealth use in India

The themes generated and their subthemes are mapped in Fig. 3. The relationships between the subthemes are described in Table 1.

**Table 1:** Relationships between subthemes that influenced mHealth use in India

**Environment Subthemes**	Influence	Subthemes affected	Exemplary References
Availability of reliable health information	Increases or decreases the need to access information through credible sources like mHealth systems	Users’ needs, desires and fears	[51], [52]
Family	determined CHWs and beneficiaries’ access to mHealth systems through sharing devices	Users’ access to mobile phones	[32, 43–45, 50]
	Provided affirmation to CHWs who used mHealth	Users’ needs, desires and fears	[53]
	Caused data errors	Users’ experiences of being helped or hindered by mHealth	[46]
	Reduced confidentiality through sharing phones	mHealth system Protection of privacy	[47]
Community	Social norms influenced the trust beneficiaries had in information provided by mHealth system	Users’ needs, desires and fears	[44]
	Stigma forced users to conceal mHealth use	Users’ needs desires and fears	[40, 52]
	Social norms hindered the availability of beneficiaries to CHWs for mHealth interventions	Users’ ability to use mHealth	[36, 38]
Mobile network	Free outgoing calls facilitated engagement with beneficiaries	Users’ experiences of being helped or hindered by mHealth	[41]
	Poor network hindered mHealth systems from working properly	Mhealth system usability	[36 – 41]
Health system	Lack of reimbursement of costs of mHealth demotivated users	Users’ experiences of being helped or hindered by mHealth	[47]
	Pressure from supervisors and incentives motivated users	Users’ experiences of being helped or hindered by mHealth	[45]
	Peer support helped users learn to use mHealth systems	Users’ ability to use mHealth	[49, 50]
**mHealth system subthemes**			
Protection of privacy	Users feared using mHealth would disclose their personal information to persons offline	Users’ needs, desires and fears	[40, 52]
	Ensuring privacy online determined whether beneficiaries were willing to receive benefits from an mHealth system	Users’ experiences of being helped or hindered by mHealth	[54]
Usability	Characteristics of health information determined how acceptable and effective health information messages were	Users’ experiences of being helped or hindered by mHealth	[51, 52, 58, 59]
	Some systems were considered easy to use while others were difficult and confusing.	Users’ ability to use mHealth	[40, 42, 44–47, 49, 53, 54, 58]
	Some systems were useful	Users’ experience of being helped or hindered by mHealth	[44, 49, 56]
	Having a simple interface, local languages, using visual media, and having work arounds made it easier to use mHealth	Users’ ability to use mHealth	[37, 38, 42, 44, 46–48, 51, 52, 54, 59]
Technology barriers	Technology barriers inhibited mHealth use	Users’ ability to use mHealth	[36, 38, 40, 42]
Technical support	Influenced whether users could use mHealth	Users’ ability to use mHealth	[40, 42, 45, 51]

The data indicates that mHealth systems are adopted when the environment supports its use; when users are motivated and have the ability to use mHealth systems; and when mHealth systems are aligned with the environmental context and fulfill users’ needs and desires.

## DISCUSSION

### Interpretation

The data were interpreted through theories of technology adoption – the Technology Acceptance Model [11], the Unified Theory of Adoption and Use of Technology [12] and the Updated DeLone and Maclean model of information system success [10] to explain the effects of perceived phenomena on mHealth use. The themes Environment, User and mHealth system generated from the data were informed by constructs in frameworks for health IT systems by Abejirinde et al. [60], Johnson, Johnson and Zhang [61] and Lehmann [18]. The themes are closest to the approach by Johnson, Johnson and Zhang [61] that considered environmental, user, and task-related effects on information system use for health information system design and optimization.

The findings among CHWs in the dataset were similar to those found in other reviews of mHealth use for primary healthcare in resource constrained settings [62–64]. This study is unique in exploring health system beneficiaries’, clinicians and tertiary hospital staff perceptions and experiences in Indian contexts.

### Implications

The findings of the review have implications for mHealth design, development, and implementation in India. Heeks [65] proposed that changes are required in health information system design and development to align health information systems with realities of the settings they are deployed to avoid system failure. Concurrently, changes in the contextual realities are required to improve healthcare outcomes. This study elicited the dimensions along which mHealth system design needs to be aligned with environments and users of mHealth systems, uncovering potential design reality gaps in mHealth systems in India. The data demonstrated the considerable variation in the influences on mHealth use across settings and health programs in India. Therefore, achieving alignment between mHealth systems and the environment and users requires research into the environment and users of a proposed system and using this research to guide design and development. This requires a human-centered design approach to mHealth before and during system development and for system optimization after deployment. The design reality gaps elicited in the data also point to the need for bringing the reality of Indian contexts closer to mHealth design. The data uncovered the effects of the digital divide on mHealth use in India due to gender and other socio-economic disparities, in keeping with the literature on the Indian digital divide [66–68]. Beneficiaries and healthcare providers from disadvantaged communities do not have sufficient access to digital technology and digital literacy to realize the potential that mHealth systems hold for them. Even if access to technology improves as technology becomes more affordable, their needs for digital literacy education will have to be addressed effectively and at scale to include them in India’s digital health ecosystem. Without closing the digital divide, mHealth systems are likely to fail the communities that need them the most. In terms of implementation, the data indicates that implementations among disadvantaged communities will require effective training and an incremental approach as sudden big changes in systems are likely to exceed the capacity of the environment and users to adapt to new systems.

A major limitation of this study is that the screening and appraisal of studies by the reviewers were carried out sequentially. This increases the risk of bias in selecting studies and the risk of missing relevant studies.

## CONCLUSION

mHealth system adoption in India is influenced by the interactions between environmental, user-related and mHealth system related factors. Alignment between these factors determines the adoption of mHealth systems. To realize the potential that mHealth systems hold for Indian health systems, the design reality gaps between mHealth systems and their environments and users need to be addressed by contextualization through human-centered design, effective system support and training, and by addressing the digital divide in India.

## Supplementary Material

appendices_2024-11-13_oqae046

## Data Availability

The data underlying this article will be shared on reasonable request to the corresponding author.
