# Supplementary material for: A systematic review and qualitative evidence synthesis of factors affecting mHealth adoption in India
Source: Oxf Open Digit Health. 2024 Nov 19;2:oqae046. doi: 10.1093/oodh/oqae046 (PMC11998589; doi:10.1093/oodh/oqae046)
Supplement: appendices_2024-11-13_oqae046 [file appendices_2024-11-13_oqae046.docx]

Supplementary Materials

# A Systematic Review and Qualitative Evidence Synthesis of factors affecting mHealth adoption in India

Verghese Thomas*, Judy Jenkins, Jomin George

## Author affiliations:

Verghese Thomas*

- Institution 1: Health Data Science, Swansea University, Singleton Park, Swansea, Wales
- Institution 2: Division of Medical Informatics, St John’s Research Institute, Bangalore, India
- Address: Division of Medical Informatics, SJRI, 100 feet road, John Nagar, Koramangala, Bangalore, Karnataka 560034
- Email: 2030498@swansea.ac.uk

Judy Jenkins

- Institution: Health Data Science, Swansea University, Singleton Park, Swansea Wales

Jomin George

- Institution: Health Data Science, Swansea University, Singleton Park, Swansea Wales

# Appendix 1 Characteristics of Studies examined

All studies examined are from India

| Name | Location in India | | | Study years | | Data collection | | Participants (number) | Health issue | | mHealth system | | WHO digital health intervention classification 2023 | |
| --- | --- | --- | --- | --- | --- | --- | --- | --- | --- | --- | --- | --- | --- | --- |
| 1. Barve et al (19) | Rural Pune district | | | 2020 | | In-depth interviews | | Outpatients (15)  App users (15) | COVID-19 | | Aarogya Setu | | 2.2.1 | |
| 1. Bhat et al (55) | Anekal, Bangalore  (rural) | | | Not stated | | Focus group discussions | | Women with major depressive disorder (69) | Mental health | | Formative research on mHealth, no specific system | | Not applicable | |
| 1. Bhatt et al (53) | -Vellore, Tamil Nadu  -Mungeli, Chhattisgarh  -Padhar, Madhya Pradesh  (all rural) | | | 2016-2018 | | Focus group discussions In-depth interviews | | Nurses (2)  Nurse assistants (2)  CHWs (5)  Program administrator (1) | Cancer | | SIM app | | 2.3.3 | |
| 1. Bhavnani et al (20) | Rewari, Haryana (rural) | | | 2018 | | In-depth interviews | | Mothers of toddlers (9) | Child development | | Developmental assessment on an E-Platform (DEEP) | | 2.3.3 | |
| 1. Bondre et al (54) | Bhopal, Madhya Pradesh (urban) | | | 2020 – 2021 | | Focus group discussions | | Patients with schizophrenia (11)  Caregivers of patients (14)  Mental health clinicians (19) | Mental health | | Formative research on mHealth apps, no specific system | | Not applicable | |
| Name | | Location in India | Study years | | Data collection | | Participants (number) | | | Health issue | | mHealth system | | WHO classification |
| 1. Chan et al (28) | | Pune, Maharashtra  (urban) | 2018 | | In-depth interviews | | Female patients with asthma (3)  Male patients with asthma (4) | | | Asthma | | ASTHMAXcel | | 1.6.1 |
| 1. Charanthimath et al (42) | | Karnataka  (rural) | 2017 | | Qualitative survey | | ASHAs* (24)  ANMs** (24) | | | Maternal and child health | | Piers On the Move | | 2.3.1 |
| 1. Gonsalves et al (58) | | Goa (urban) | 2019 – 2020 | | In-depth interviews | | Secondary school students (22) | | | Mental health | | POD Adventures | | 1.1.2 |
| 1. Gopalakrishnan et al (36) | | -Samastipur Bihar  -Ujjain, Madhya Pradesh  (all rural) | 2018 | | In-depth interviews | | Anganwadi Workers (32)  Pregnant women (25)  Lactating women (30) | | | Maternal and child health | | Common Application Software | | 1.1.2, 2.2.1, 2.2.4, 2.3.1 |
| 1. Hazra, Khan and Mondal (29) | | Jhansi,  Uttar Pradesh (rural) | 2014 | | In-depth interviews  Focus group discussions | | Husbands of pregnant women (10)  ASHAs | | | Maternal and child health | | IVRS audio messages | | 1.1.2 |
| 1. Ilozumba et al (38) | | Deoghar, Jharkhand  (rural) | 2015-2016 | | In-depth interviews  Focus group discussions | | Women (12)  Men (16)  CHWs (16) | | | Maternal and child health | | Mobiles for Mothers | | 1.1.2, 2.2.1 |
| 1. Ismail and Kumar (41) | | Delhi  (urban) | 2016-2018 | | In-depth interviews  Participant observations  Text analysis of WhatsApp group chats | | ASHAs (20) | | | General use for supporting work in healthcare | | WhatsApp use by ASHAs | | 2.5.5 |
| 1. Jose et al (30) | | Kerala | Not stated | | In-depth interviews  Focus group discussion | | Subject experts (5)  Consultants (6)  Primary care physicians (8)  Doctors from different administrative levels (18) | | | Cancer | | M-OncoEd | | 2.8.1 |

*ASHAs – Accredited Social Health Activists (CHWs) ** ANMs – Auxiliary Nurse Midwives (public health nurses)

| Name | Location in India | Study years | Data collection | Participants (number) | Health issue | mHealth system | WHO classification |
| --- | --- | --- | --- | --- | --- | --- | --- |
| 1. Kodali and Das (56) | Krishna District,  Andhra Pradesh (rural) | 2018-2019 | In-depth interviews | ANMs**(12)  Planners (9) | All public health programs | All public health program apps | 2.2.1, 2.2.2, 2.2.3, 2.2.4 |
| 1. Mehta et al (47) | Vadodara,  Gujarat  (urban) | 2016 – 2017 | In-depth interviews  Focus group discussions | Counsellors (16)  Program managers (2)  People living with HIV (4) | HIV | M-Track | 2.2.1 |
| 1. Nagraj et al (49) | Jhajjar, Haryana and Guntur, Andhra Pradesh  (rural) | 2019-2020 | In-depth interviews  Focus group discussions | CHWs (56)  Pregnant or postpartum women (5) | NCDs and Maternal and child health | SMARThealth Pregnancy | 2.2.2, 2.3.1 |
| 1. Okolo et al (50) | Uttar Pradesh  (rural) | 2020 | In-depth interviews, online | ASHAs*(21) | Maternal child health | Formative research on mHealth apps. Used video provocation of an AI enabled app being used by a CHW | Not applicable |
| 1. Pahwa et al (48) | Delhi  (urban) | 2016 | In-depth interviews  Text analysis | Doctors (5)  Nurses (4)  Technician (1)  Quality improvement coach (1) | Neonatal health | WhatsApp group forum use by neonatal ICU team | 2.5.5 |
| 1. Pai and Alathur (24) | Karnataka | 2017 | In-depth interviews | General population (59)  Healthcare provider (1) | No specific health issue studied | Formative study on use of mobile phones | Not applicable |

*ASHAs – Accredited Social Health Activists (CHWs) ** ANMs – Auxiliary Nurse Midwives (public health nurses)

| Name | Location in India | Study years | Data collection | Participants (number) | Health issue | mHealth system | WHO classification |
| --- | --- | --- | --- | --- | --- | --- | --- |
| 1. Panda et al (59) | Behrampur,  Odisha  (urban) | 2020 | In-depth interviews  Focus group discussions | Persons using smokeless (26)  Primary care physicians (5)  Counsellors (12) | Tobacco cessation | Formative research on mHealth apps. | Not applicable |
| 1. Pendse et al (25) | Punjab  (rural) | 2020-2021 | Secondary analysis of intervention pilot data | Early postpartum women (44) | Maternal and child health | Formative research on mobile phone use | Not applicable |
| 1. Prakash et al (26) | Multiple cities (urban) | Not stated | Survey | Non adopters of Digital Contact Tracing App | COVID-19 | Aarogya Setu app use | 2.2.1 |
| 1. Rawat et al (52) | Mumbai  (urban) |  | In-depth interviews | Men who have sex with men (24)  Hijras (third gender individuals) (4)  Health workers (10)  mHealth developers (8) | Sexually transmitted infections | Formative research on mHealth apps. | Not applicable |
| 1. Saha et al (27) | Devbhumi Dwarka and Panchmahal, Gujarat  (rural) | 2019-2020 | Survey | ANMs** (29)  Data entry operators (120  Medical officers (10) | Maternal and child health | TeCHO+ app | 2.2.1, 2.2.2 |
| 1. Sampathkumar et al (28) | Chennai  (urban) | 2018-2019 | Focus group discussions | Postnatal women (6) | Maternal and child health | Text messaging service for postnatal care | 1.1.2 |

*ASHAs – Accredited Social Health Activists (CHWs) ** ANMs – Auxiliary Nurse Midwives (public health nurses)

| Name | Location in India | Study years | Data collection | Participants (number) | Health issue | mHealth system | WHO classification |
| --- | --- | --- | --- | --- | --- | --- | --- |
| 1. Sarin et al (29) | Two districts of Uttar Khand and 3 districts of Jharkhand  (rural) | 2020 | Interviews | ANMs** and staff nurses (124) | Maternal and child health | Safe Delivery App | 2.8.1 |
| 1. Scott et al. (44) | Madhya Pradesh (rural) | 2019 | In-depth interviews | Women eligible for IVRS calls (29)  Husbands of women eligible for IVRS calls (30)  Other family members of women (23) | Maternal and child health | Kilkari | 1.1.2 |
| 1. Scott et al (45) | Ajmer, Sikar, and Pali,  Rajasthan  (rural) | 2018 | In-depth interviews  Focus group discussions | ASHAs* (30)  ANMs** (6)  ASHA supervisors (6)  Block ASHA coordinators (3)  District officers (3)  State officials (2) | mLearning system | Mobile Academy | 2.8.1 |
| 1. Seshu et al (37) | Samastipur, Bihar  (rural) | Not stated | In-depth interviews  Focus group discussions | Women with postnatal depression (20) | Mental health | IVRS based messaging system | 1.1.2 |

*ASHAs – Accredited Social Health Activists (CHWs) ** ANMs – Auxiliary Nurse Midwives (public health nurses)

| Name | Location in India | Study years | Data collection | Participants (number) | Health issue | mHealth system | WHO classification |
| --- | --- | --- | --- | --- | --- | --- | --- |
| 1. Singh, Sudarshan and Tandon (30) | Karnataka  (rural) | Not stated | In-depth interviews | health care providers using DAKSH to monitor labour in rural health centres in India (number not stated) | Maternal and child health | DAKSH: Digital Partograph and Intrapartum Monitoring Mobile Application | 2.10.3 |
| 1. Deb et al (51) | New Delhi  (urban) | 2016-2017 | Focus group discussions | Caregivers of patients with mental health conditions (15)  Health care providers (7) | Mental health | Formative research on mHealth apps. | Not applicable |
| 1. Suryavanshi et al (31) | Pune, Sangli, Satara, and Thane,  Maharashtra  (urban and rural) | 2015-2017 | In-depth interviews | Community health workers (15)  HIV infected pregnant and breastfeeding women | HIV prevention and Maternal and child health | Electronic Mobile Comprehensive Health Application | 2.8.1 |
| 1. Tewari et al (43) | West Godavari,  Andhra Pradesh  (rural) | 2016-2017 | In-depth interviews  Focus group discussions | Community members (125)  ASHAs (19)  Project field workers (16)  Primary health centre doctors (5)  Government official (1) | Mental health | SMART Mental health programme | 2.3.1 |

| Name | Location in India | Study years | Data collection | Participants (number) | Health issue | mHealth system | WHO classification |
| --- | --- | --- | --- | --- | --- | --- | --- |
| 1. Thomas et al (46) | Chennai,  Vellore, and  Mumbai  (urban and rural) | 2017-2018 | In-depth interviews | Patients with TB (32)  Patients with TB and HIV (30)  Health care providers (31) | Infectious diseases | 99 DOTS | 2.4.2 |
| 1. Thomas et al (40) | Chennai and Mumbai  (urban) | 2017-2018 | In-depth interviews | Patients with MDR TB (65)  Health care providers (10)_ | Infectious diseases | Medication Event Reminder Monitor | 1.1.3 |
| 1. Usmanova et al (32) | Madhya Pradesh and Rajasthan  (rural) | 2020 | In-depth interviews | Medical officers (9)  Labor room supervisors (10)  Staff nurses (25) | Maternal and child health | ASMAN | 2.2.2, 2.2.3, 4.1.3, 2.8.1, 2.3.1, 2.4.4 |
| 1. Venkataraghavan et al (33) | Udipi, Karnataka (rural) | 2020 | In-depth interviews | Medical officers (15) | All public health programs | All public health program apps | 2.2.1, 2.2.2, 2.2.3, 2.2.4 |
| 1. Williams et al (34) | Ludhiana,  Punjab (urban) | 2020 | Survey | Medical students (80)  Medical college faculty (4) | Dermatology | WhatsApp forum used as a learning intervention | 2.5.5 |
| 1. Yadav et al (35) | New Delhi  (urban) | 2020-2021 | Survey | Patients using a telemedicine service (201) | Dermatology | WhatsApp calls for tele dermatology | 2.4.1 |

# Appendix 2 Appraisal of studies using CASP (2018)

|  | 1. Was there a clear statement of the aims of the research? | 2. Is a qualitative methodology appropriate? | 3. Was the research design appropriate to address the aims of the research? | 4. Was the recruitment strategy appropriate to the aims of the research? | 5. Was the data collected in a way that addressed the research issue? | 6. Has the relationship between researcher and participants been adequately considered? | 7. Have ethical issues been taken into consideration? | 8. Was the data analysis sufficiently rigorous? | 9. Is there a clear statement of findings? | 10. How valuable is the research? | Included in the synthesis? |
| --- | --- | --- | --- | --- | --- | --- | --- | --- | --- | --- | --- |
| 1. Barve et al (19) | **Yes** | **Yes** | **Can't Tell** | **No** | **Can't Tell** | **No** | **Can't Tell** | **No** | **No** | **No** | **No** |
| 1. Bhat et al (55) | **Yes** | **Yes** | **Yes** | **Yes** | **Yes** | **Yes** | **Yes** | **Yes** | **Yes** | **Yes** | **Yes** |
| 1. Bhatt et al (53) | **Yes** | **Yes** | **Yes** | **Yes** | **Yes** | **Can't Tell** | **Yes** | **Yes** | **Yes** | **Yes** | **Yes** |
| 1. Bhavnani et al (20) | **Yes** | **Yes** | **No** | **Can't Tell** | **Yes** | **No** | **Yes** | **Can't Tell** | **Yes** | **Can't Tell** | **No** |
| 1. Bondre et al (54) | **Yes** | **Yes** | **Yes** | **Yes** | **Yes** | **Yes** | **Yes** | **Yes** | **Yes** | **Yes** | **Yes** |
| 1. Chan et al (28) | **Yes** | **No** | **Yes** | **Yes** | **Can't Tell** | **Can't Tell** | **Yes** | **No** | **No** | **Can't Tell** | **No** |
| 1. Charanthimath et al (42) | **Yes** | **Yes** | **Yes** | **Yes** | **Yes** | **Yes** | **Yes** | **Yes** | **Yes** | **Yes** | **Yes** |

|  | 1. Was there a clear statement of the aims of the research? | 2. Is a qualitative methodology appropriate? | 3. Was the research design appropriate to address the aims of the research? | 4. Was the recruitment strategy appropriate to the aims of the research? | 5. Was the data collected in a way that addressed the research issue? | 6. Has the relationship between researcher and participants been adequately considered? | 7. Have ethical issues been taken into consideration? | 8. Was the data analysis sufficiently rigorous? | 9. Is there a clear statement of findings? | 10. How valuable is the research? | Included in the synthesis? |
| --- | --- | --- | --- | --- | --- | --- | --- | --- | --- | --- | --- |
| 1. Gonsalves et al (58) | **Yes** | **Yes** | **Yes** | **Yes** | **Yes** | **Can't Tell** | **Yes** | **Yes** | **Yes** | **Yes** | **Yes** |
| 1. Gopalakrishnan et al (36) | **Yes** | **Yes** | **Yes** | **Yes** | **Yes** | **Yes** | **Yes** | **Yes** | **Yes** | **Yes** | **Yes** |
| 1. Hazra, Khan and Mondal (29) | **Yes** | **Yes** | **Yes** | **Can't Tell** | **Yes** | **No** | **Yes** | **No** | **Yes** | **Yes** | **No** |
| 1. Ilozumba et al (38) | **Yes** | **Yes** | **Yes** | **Yes** | **Yes** | **Yes** | **Yes** | **Yes** | **Yes** | **Yes** | **Yes** |
| 1. Ismail and Kumar (41) | **Yes** | **Yes** | **Yes** | **Yes** | **Yes** | **Yes** | **Yes** | **Yes** | **Yes** | **Yes** | **Yes** |
| 1. Jose et al (30) | **Yes** | **Yes** | **Yes** | **Yes** | **Yes** | **Yes** | **Yes** | **Yes** | **Yes** | **Yes** | **No*** |
| 1. Kodali and Das (56) | **Yes** | **Can't Tell** | **Can't Tell** | **Yes** | **Can't Tell** | **No** | **Yes** | **Yes** | **Yes** | **Yes** | **Yes** |
| 1. Mehta et al (47) | **Yes** | **Yes** | **Yes** | **Yes** | **Yes** | **Yes** | **Yes** | **Yes** | **Yes** | **Yes** | **Yes** |

*Does not have a conflict-of-interest statement

|  | 1. Was there a clear statement of the aims of the research? | 2. Is a qualitative methodology appropriate? | 3. Was the research design appropriate to address the aims of the research? | 4. Was the recruitment strategy appropriate to the aims of the research? | 5. Was the data collected in a way that addressed the research issue? | 6. Has the relationship between researcher and participants been adequately considered? | 7. Have ethical issues been taken into consideration? | 8. Was the data analysis sufficiently rigorous? | 9. Is there a clear statement of findings? | 10. How valuable is the research? | Included in the synthesis? |
| --- | --- | --- | --- | --- | --- | --- | --- | --- | --- | --- | --- |
| 1. Nagraj et al (49) | **Yes** | **Yes** | **Yes** | **Yes** | **Yes** | **Can't Tell** | **Yes** | **Yes** | **Yes** | **Yes** | **Yes** |
| 1. Okolo et al (50) | **Yes** | **Yes** | **Yes** | **Yes** | **Yes** | **Yes** | **Yes** | **Yes** | **Yes** | **Yes** | **Yes** |
| 1. Pahwa et al (48) | **Yes** | **Yes** | **Yes** | **Yes** | **Yes** | **No** | **Yes** | **Yes** | **Yes** | **Yes** | **Yes** |
| 1. Pai and Alathur (24) | **Can't Tell** | **Can't Tell** | **Can't Tell** | **Can't Tell** | **Can't Tell** | **No** | **No** | **No** | **Can't Tell** | **Can't Tell** | **No** |
| 1. Panda et al (59) | **Yes** | **Yes** | **Yes** | **Yes** | **Yes** | **Can't Tell** | **Yes** | **Yes** | **Yes** | **Yes** | **Yes** |
| 1. Pendse et al (25) | **Yes** | **Yes** | **Can't Tell** | **Yes** | **Yes** | **No** | **Can't Tell** | **Can't Tell** | **Yes** | **Yes** | **No** |
| 1. Prakash et al (26) | **Yes** | **Can't Tell** | **Can't Tell** | **Yes** | **Can't Tell** | **No** | **No** | **Can't Tell** | **Yes** | **Yes** | **No** |
| 1. Rawat et al (52) | **Yes** | **Yes** | **Yes** | **Yes** | **Yes** | **Can't Tell** | **Yes** | **Yes** | **Yes** | **Yes** | **Yes** |
| 1. Saha et al (27) | **Yes** | **Can't Tell** | **Yes** | **Can't Tell** | **Yes** | **No** | **Yes** | **Can't Tell** | **Can't Tell** | **Yes** | **No** |

|  | 1. Was there a clear statement of the aims of the research? | 2. Is a qualitative methodology appropriate? | 3. Was the research design appropriate to address the aims of the research? | 4. Was the recruitment strategy appropriate to the aims of the research? | 5. Was the data collected in a way that addressed the research issue? | 6. Has the relationship between researcher and participants been adequately considered? | 7. Have ethical issues been taken into consideration? | 8. Was the data analysis sufficiently rigorous? | 9. Is there a clear statement of findings? | 10. How valuable is the research? | Included in the synthesis? |
| --- | --- | --- | --- | --- | --- | --- | --- | --- | --- | --- | --- |
| 1. Sampathkumar et al (28) | **Yes** | **Can't Tell** | **Yes** | **Can't Tell** | **Yes** | **Can't Tell** | **Yes** | **No** | **Yes** | **Can't Tell** | **No** |
| 1. Sarin et al (29) | **Yes** | **No** | **Can't Tell** | **Yes** | **Yes** | **No** | **Yes** | **No** | **Yes** | **Yes** | **No** |
| 1. Scott et al. (44) | **Yes** | **Yes** | **Yes** | **Yes** | **Yes** | **Can't Tell** | **Yes** | **Yes** | **Yes** | **Yes** | **Yes** |
| 1. Scott et al (45) | **Yes** | **Yes** | **Yes** | **Yes** | **Yes** | **Can't Tell** | **Yes** | **Yes** | **Yes** | **Yes** | **Yes** |
| 1. Seshu et al (37) | **Yes** | **Yes** | **Yes** | **Yes** | **Yes** | **Can't Tell** | **Yes** | **Yes** | **Yes** | **Yes** | **Yes** |
| 1. Singh, Sudarshan and Tandon (30) | **No** | **Can't Tell** | **Can't Tell** | **Can't Tell** | **No** | **No** | **Can't Tell** | **No** | **No** | **No** | **No** |
| 1. Deb et al (51) | **Yes** | **Yes** | **Yes** | **Yes** | **Yes** | **Yes** | **Yes** | **Yes** | **Yes** | **Yes** | **Yes** |
| 1. Suryavanshi et al (31) | **Yes** | **Yes** | **Yes** | **Can't Tell** | **Yes** | **Can't Tell** | **Yes** | **Yes** | **Yes** | **Yes** | **No*** |
| 1. Tewari et al (43) | **Can't Tell** | **Yes** | **Yes** | **Can't Tell** | **Yes** | **No** | **Yes** | **Yes** | **Yes** | **Yes** | **Yes** |

*Excluded because of a potential conflict of interest

|  | 1. Was there a clear statement of the aims of the research? | 2. Is a qualitative methodology appropriate? | 3. Was the research design appropriate to address the aims of the research? | 4. Was the recruitment strategy appropriate to the aims of the research? | 5. Was the data collected in a way that addressed the research issue? | 6. Has the relationship between researcher and participants been adequately considered? | 7. Have ethical issues been taken into consideration? | 8. Was the data analysis sufficiently rigorous? | 9. Is there a clear statement of findings? | 10. How valuable is the research? | Included in the synthesis? |
| --- | --- | --- | --- | --- | --- | --- | --- | --- | --- | --- | --- |
| 1. Thomas et al (46) | **Yes** | **Yes** | **Yes** | **Yes** | **Yes** | **Can't Tell** | **Yes** | **Yes** | **Yes** | **Yes** | **Yes** |
| 1. Thomas et al (40) | **Yes** | **Yes** | **Yes** | **Yes** | **Yes** | **Can't Tell** | **Yes** | **Yes** | **Yes** | **Yes** | **Yes** |
| 1. Usmanova et al (32) | **Yes** | **Yes** | **Can't Tell** | **Yes** | **Yes** | **Yes** | **Yes** | **Yes** | **Yes** | **Yes** | **No*** |
| 1. Venkataraghavan et al (33) | **Can't Tell** | **Yes** | **Can't Tell** | **Yes** | **Can't Tell** | **No** | **Can't Tell** | **Can't Tell** | **Yes** | **Yes** | **No** |
| 1. Williams et al (34) | **Yes** | **Can't Tell** | **Can't Tell** | **Can't Tell** | **No** | **No** | **Yes** | **No** | **Can't Tell** | **No** | **No** |
| 1. Yadav et al (35) | **Can't Tell** | **Can't Tell** | **Can't Tell** | **Can't Tell** | **No** | **Yes** | **Can't Tell** | **No** | **Can't Tell** | **Can't Tell** | **No** |

*Excluded because of a potential conflict of interest
